# Artificially-generated consolidations and balanced augmentation increase performance of U-net for lung parenchyma segmentation on MR images

**DOI:** 10.1371/journal.pone.0285378

**Published:** 2023-05-09

**Authors:** Cristian Crisosto, Andreas Voskrebenzev, Marcel Gutberlet, Filip Klimeš, Till F. Kaireit, Gesa Pöhler, Tawfik Moher, Lea Behrendt, Robin Müller, Maximilian Zubke, Frank Wacker, Jens Vogel-Claussen

**Affiliations:** 1 Institute for Diagnostic and Interventional Radiology, Hannover Medical School (MHH), Hannover, Germany; 2 Biomedical Research in Endstage and Obstructive Lung Disease Hannover (BREATH), Member of the German Centre for Lung Research (DZL), Hannover, Germany; University of Alberta, CANADA

## Abstract

**Purpose:**

To improve automated lung segmentation on 2D lung MR images using balanced augmentation and artificially-generated consolidations for training of a convolutional neural network (CNN).

**Materials and methods:**

From 233 healthy volunteers and 100 patients, 1891 coronal MR images were acquired. Of these, 1666 images without consolidations were used to build a binary semantic CNN for lung segmentation and 225 images (187 without consolidations, 38 with consolidations) were used for testing. To increase CNN performance of segmenting lung parenchyma with consolidations, balanced augmentation was performed and artificially-generated consolidations were added to all training images. The proposed CNN (CNN_Bal/Cons_) was compared to two other CNNs: CNN_Unbal/NoCons_—without balanced augmentation and artificially-generated consolidations and CNN_Bal/NoCons_—with balanced augmentation but without artificially-generated consolidations. Segmentation results were assessed using Sørensen-Dice coefficient (*SDC*) and Hausdorff distance coefficient.

**Results:**

Regarding the 187 MR test images without consolidations, the mean *SDC* of CNN_Unbal/NoCons_ (92.1 ± 6% (mean ± standard deviation)) was significantly lower compared to CNN_Bal/NoCons_ (94.0 ± 5.3%, *P* = 0.0013) and CNN_Bal/Cons_ (94.3 ± 4.1%, *P* = 0.0001). No significant difference was found between *SDC* of CNN_Bal/Cons_ and CNN_Bal/NoCons_ (*P* = 0.54).

For the 38 MR test images with consolidations, *SDC* of CNN_Unbal/NoCons_ (89.0 ± 7.1%) was not significantly different compared to CNN_Bal/NoCons_ (90.2 ± 9.4%, *P* = 0.53). *SDC* of CNN_Bal/Cons_ (94.3 ± 3.7%) was significantly higher compared to CNN_Bal/NoCons_ (*P* = 0.0146) and CNN_Unbal/NoCons_ (*P* = 0.001).

**Conclusions:**

Expanding training datasets via balanced augmentation and artificially-generated consolidations improved the accuracy of CNN_Bal/Cons_, especially in datasets with parenchymal consolidations. This is an important step towards a robust automated postprocessing of lung MRI datasets in clinical routine.

## Introduction

In the past years, MRI gained interest as an ionizing radiation-free alternative to computer tomography (CT) and single-photon emission computed tomography (SPECT) [1] for functional assessment of the lung. Among other Fourier Decomposition [2] based techniques such as phase-resolved functional lung (PREFUL) MR imaging allows assessment of pulmonary ventilation and perfusion dynamics in free breathing without the use of contrast agents or gas inhalation [3]. However, manual segmentation of the lung parenchyma requires time-consuming manual interaction, which impedes implementation of MR lung imaging methods, like PREFUL, into clinical workflow. Therefore, a fast and automatic lung parenchyma segmentation is desirable.

Convolutional neural networks (CNNs) have broadened the potential of computational models to represent data on multiple levels of abstraction [4] and multiple lung segmentation CNNs based on 2D and 3D data have been proposed [5–7] However, these methods have been developed and tested on limited pathologies or single cases of disease [8, 9]. Especially, accurate segmentation of lung parenchyma with peripheral consolidations, which are difficult to visually separate from surrounding tissue, is currently a challenge for each CNN in clinical usage [10].

Willers et al., presented a methodology for an automatic segmentation of lung parenchyma through a CNN [11]. In this work, a mean Sørenson-Dice coefficient (*SDC*) of 6% difference between CNN and manual segmentation was shown, which authors attributed to existing consolidations or atelectasis in the study cohort. They concluded, that these differences can generate discrepancies in functional parameters up to 2% for relative perfusion and 3% for relative fractional ventilation. Thus, severe pathologies like extensive consolidation may be segmented incorrectly and should be manually corrected [11].

However, limited availability of MR images of patients with real lung consolidations hampers the training of a network for segmenting lung parenchyma with consolidations. Therefore, the insertion of artificially-generated consolidations on MRI lung images might be a viable solution to improve automated lung segmentation similar to Vajira et al., who showed a pipeline to generate synthetic data in MR medical images for improving the segmentation results of real data [12]. In addition, Costa et al., implemented an algorithm for the creation of retinal vessel network synthetic data, which led to increased efficiency in training and segmentation accuracy [13].

Further, CNN performance can be affected by unbalanced training datasets, i.e. an under- or overrepresentation of a specific image kind (e.g. images from one MR sequence or of one slice position). To overcome this problem, conventional data augmentation has recently been utilized in different application areas [14, 15]. Mariani et al., proposed a model for balancing datasets [16] and the results showed that when generating images for the minority classes the training performance increased as well as the classification. Further, Gao et al. implemented an augmentation method for unbalanced datasets [17]. This technique includes an augmentation through deletion, and sentence translation for the minority classes, which resulted in balanced datasets.

The aim of this work is to improve automated lung segmentation especially in difficult cases with consolidations, and to examine the effect of expanding a training dataset for U-net CNN training with artificially-generated consolidations and balanced augmentation by comparing the accuracy of the resulting CNNs on the task of lung segmentation using MR images with and without lung consolidations.

## Methods

Data from different completed and ongoing studies [6, 18] of different lung diseases were collected in order to obtain a heterogenous data set. A total of 233 healthy volunteers (134 female, 99 male, mean age 62.4 ± 7.1 years) and 100 patients (56 female, 44 male, mean age 52 ± 8.2 years) were included. The patient cohort included subjects with different pathologies: chronic obstructive pulmonary disease (COPD) (n = 20 completed study [19]; 34 ongoing study; 34 female, 20 male), cystic fibrosis (CF) (n = 14 completed study [20]; n = 21 ongoing study; 20 female, 15 male) and chronic thromboembolic pulmonary hypertension (CTEPH) (n = 6 completed study; n = 5 ongoing study; 6 female, 5 male). All patients provided written informed consent.

### Image acquisition

Data acquisition was performed using the following sequences and scanners:

• *Fast Low-Angle Shot* (*FLASH) data*:

256 patients and healthy volunteers underwent MR data acquisition on a 1.5 T MR-scanner (Magnetom Avanto, Siemens Healthcare, Erlangen, Germany) in head-first supine position and with the following sequence parameters: field of view 50 x 50 cm^2^, matrix size of 128 x 128 (interpolated to 256 x 256), slice thickness 15 mm, echo time 0.67 − 0.91 ms, repetition time 1.88 − 3 ms, flip angle 4° − 8° and bandwidth 651 − 1502 Hz/pixel. Between one and nine coronal slices were acquired for each subject. Thus, a total of 1497 slices were acquired.

Further, 33 patients and healthy volunteers received FLASH imaging on a 3 T MR-scanner (Signa Premier, GE Medical System, Waukesha, WI, USA) in head-first supine position with the following settings: field of view 50 x 50 cm^2^, matrix size of 128 x 128 (interpolated to 256 x 256), slice thickness 8 − 15 mm, echo time 0.73 − 0.95 ms, repetition time 2.3 − 2.6 ms, flip angle of 5° and bandwidth 954 − 1565 Hz/pixel. Between one and three coronal slices were acquired for each subject. Thus, a total of 91 slices were acquired.

• *Balanced steady-state free precession (bSSFP) data*:

These MRI images were acquired from 35 patients and healthy volunteers on a 1.5 T MR-scanner (Magnetom Avanto, Siemens Healthcare, Erlangen, Germany) with the following sequence settings: field of view 50x50 cm^2^, matrix size of 128 x 128 (interpolated to 256 x 256), slice thickness 15 mm, echo time 0.38 − 0.81, ms, repetition time 1.08 − 1.55 ms, flip angle 27.5° − 35° and bandwidth 1490 − 2055 Hz/pixel. Between one and eleven coronal slices were acquired for each subject. Thus, a total of 303 slices were acquired.

The MR images were acquired during free breathing and all images of each slice (between 1 and 11 slices and 200–250 MR images per slice) were registered towards an intermediate respiratory state using Advanced Normalization Tools [21]. The registered images of each slice were averaged in the time domain resulting in a single averaged image per slice. Afterwards, from 233 healthy volunteers and 100 patients a total of 1891 averaged coronal MR images were manually segmented by a scientist with two years’ experience in lung MRI (C.C.) supervised by a radiologist (J.V.C., >18 years of MRI experience).

**Fig 1:**
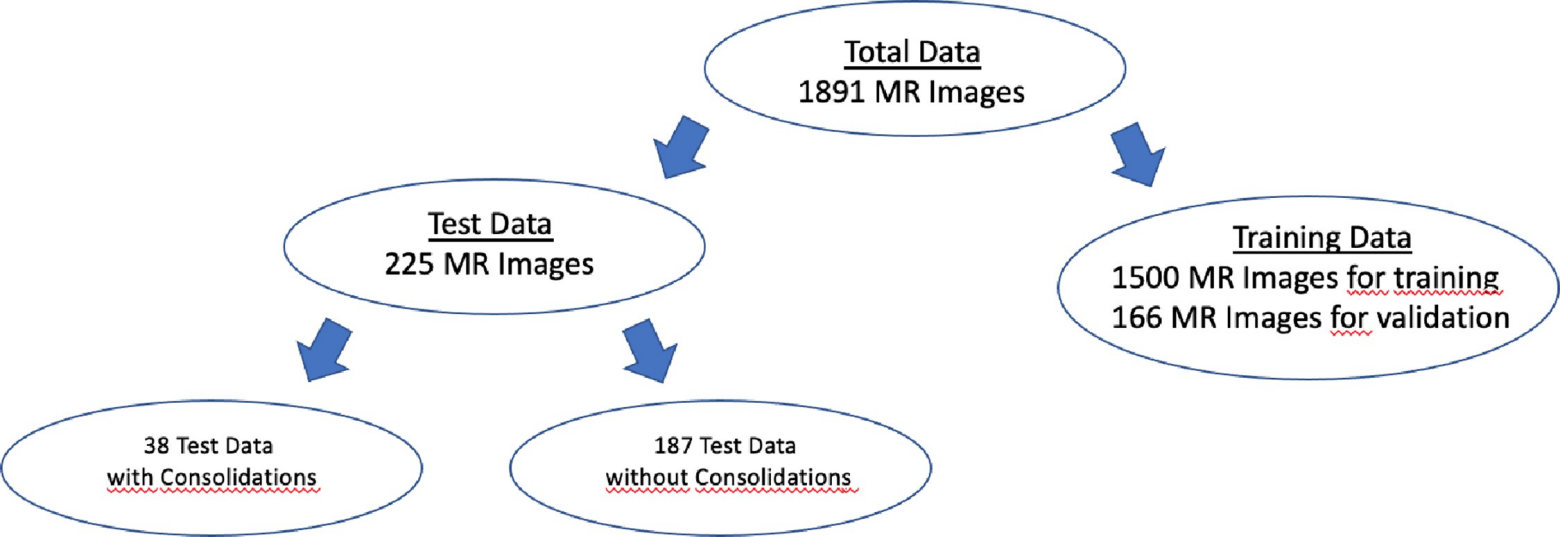
Workflow of the total data for the proposed CNN. The total data consisted of 1891 MR images. The test data was divided into two; 38 MR images with consolidations and 187 MR images without consolidations. Finally, the total training dataset contained 1666 MR images.

From these images, 1666 images were used for further analysis. Thus, after augmentation (a total of 17129 images), 10% of the data were taken for validation during training. Therefore, 225 images (187 without consolidations from healthy volunteers and 38 with consolidations from CF and COPD patients) were used for testing. See Fig 1.

In addition, we want to analyze the lung segmentation with consolidations segmented with the proposed network using the PREFUL [3] algorithm.

### Image analysis and balanced augmentation

**Fig 2:**
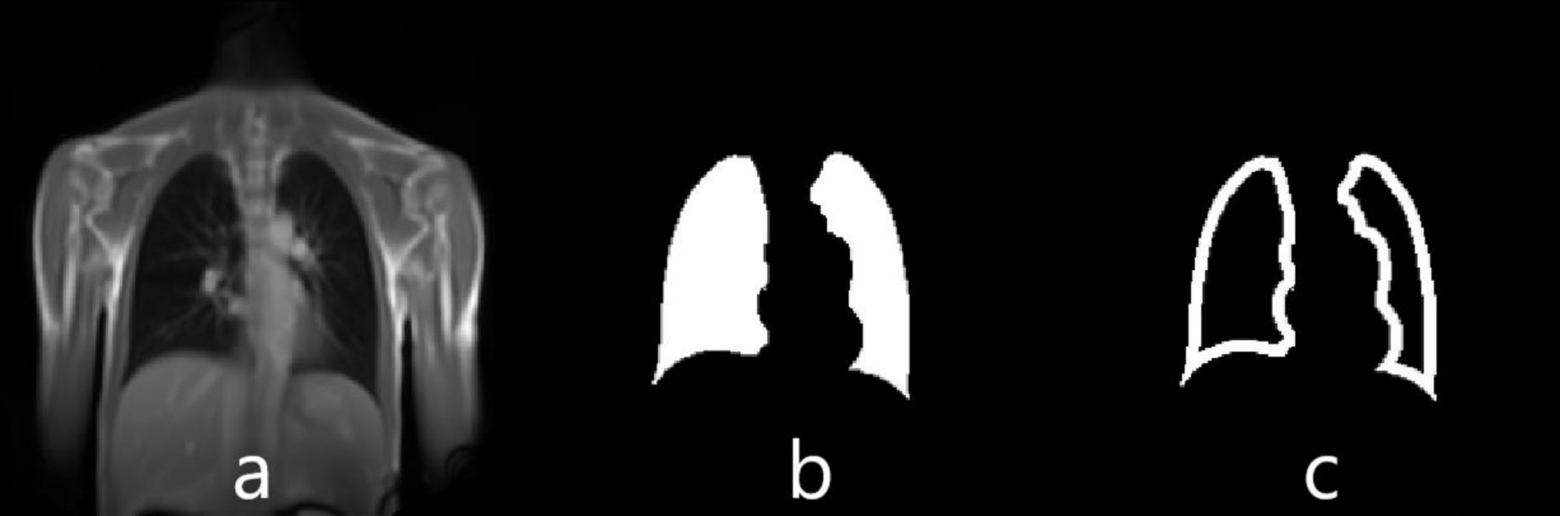
Creation of the two classes of the proposed CNN. (a) representative morphological temporally-averaged MR image, with respective manual segmentation of lung parenchyma in (b). Finally, (c) shows the two classes (ground truth) for the training of the CNN: background (black colour outside and within the lung) and lung’s contour (white colour). The contour has a width of 6 pixels.

First, from the manually segmented images the marginal voxels (width of 6 voxel) were determined to form a closed lung contour, see Fig 2C. These contours of the lungs were used as ground truth during training of the CNN. The CNN uses two classes: “contour of the lungs” (ground truth) and “background” (outside and within the lung), see Fig 2.

96% of the 2D MR datasets in our database are coronal slices centered to the trachea (ventral-dorsal direction). As a result, posterior and anterior slices (distance range: 17–60 mm from central slice) are underrepresented. For this reason, this study introduces a balanced augmentation with different augmentation factors to balance the slice positions in terms of their ventral-to-dorsal location. Thus, by multiplying different factors, each slice position has a similar number of temporally-averaged MR images during the training stage, see Table 1.

**Table 1:** Balanced augmentation. Training data split between 4 different categories: Fast Low-Angle Shot (FLASH), Anterior_Posterior_FLASH, balanced steady-state free precession (bSSFP) and Anterior_Posterior_ bSSFP. To balance the training i.e., to relatively obtain the same number of MR images, different factors were chosen. Altogether a total of 17129 MR images with their corresponding 17129 ground truth images were generated for training the CNN.

Categories	Images (n)	Augmentation Factor	Images after augmentation (in [%])
FLASH	1371	3	4113 [24.0%]
Anterior_Posterior_FLASH	21	206	4326 [25.3%]
bSSFP	256	17	4352 [25.4%]
Anterior_Posterior_ bSSFP	18	241	4338 [25.3%]
**Total**	**1666**		**17129**

Overall, 1371 FLASH images, 21 Anterior_Posterior_FLASH images, 256 bSSFP images and 18 Anterior_Posterior_bSSFP images were considered for balanced augmentation. The augmentation of the datasets consisted of the re-scaling [0.3, 0.75], X-translation [10 pixels, 20 pixels], Y-translation [10 pixels, 20 pixels] and image rotation [0°, 360°] in the plane. In addition, the ground truth images were augmented at the same time according to their corresponding MR image.

### Artificial consolidations

We define consolidation as established in The Glossary of Terms for Thoracic Imaging of the Fleischner Society [22]:

“Pathology. Consolidation refers to an exudate or other product of disease that replaces alveolar air, rendering the lung solid (as in infective pneumonia).”

Thus, due to the fact, that there are not enough real consolidations in our database, we created artificial consolidations on all 17129 MR images to train the proposed network. An experienced radiologist (J.V.C. > 18 years of experience) visually controlled the artificially-generated consolidations on the MR images.

**Fig 3:**
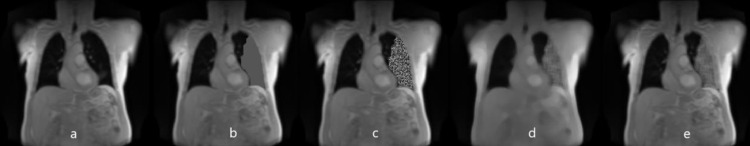
Artificially-generated consolidations. (a) shows the original morphological MR image. (b) shows the artificially-generated consolidation shape overlapped on the MR image. (c) shows the randomly filled shape with a uniformly distributed random function between the minimum and the maximum value of the lung intersecting the manually segmented lungs including vessels and airways. (d) shows image c after the 2D-median filtering. (e) shows the final visualization result of the artificially-generated consolidation algorithm after application of the 2D Gaussian filter.

The algorithm to generate artificial consolidations is outlined in the following, see also Fig 3.

1. A region inside one of the lungs is randomly selected to be transformed into an artificial consolidation see Fig 3B.
2. The region inside the lung is randomly filled with a uniformly distributed random values between the minimum and maximum value of the selected lung, see Fig 3C.
3. A 2D-median filtering function (10x10) is applied to the whole MR image, see Fig 3D.
4. Finally, by taking only the lung area (including consolidation) of step 3 and applying a 2D Gaussian filter (5x5) to the border of the artificially-generated consolidation and the MR image, the final result is obtained, see Fig 3E.

To accurately segment lung parenchyma with peripheral consolidation, during the training phase, artificial consolidations were placed on the border of each lung. Finally, the input data of this work were the morphological MR images with the artificially generated consolidations. As labels, the contours of the lung parenchyma were acquired. The output result of the CNN was the probability matrix of the lung parenchyma’s contour.

### CNN architecture

For the training, a 2D U-Net [23] architecture implemented in TensorFlow [24] was used in this work. The input layer was resized to 256 x 256 and in combination with upsampling operations resulted an output layer size of 256 x 256. The binary cross entropy function was minimized during the training process and as activation layers rectified linear units (ReLU), or ReLUs, defined as: ReLU(z) = max(0,z) [25] were selected. Adaptive Moment Estimation (Adam) [26] was chosen as a stochastic optimization method, the learning rate was 0.001, early stopping was performed with a patience of 10 epochs, the batch size was 64, 100 epochs and accuracy was chosen as metric. Finally, the training was performed on a server with a X11DPG-QTMainboard, LGA 3647, 2 x Xeon Platinum 8176 2.1GHz 56-Core, 256GB RAM, 2 x NVIDIA Quadro RTX 6000 24 GB and was computed in 3 hours.

### Postprocessing

**Fig 4:**
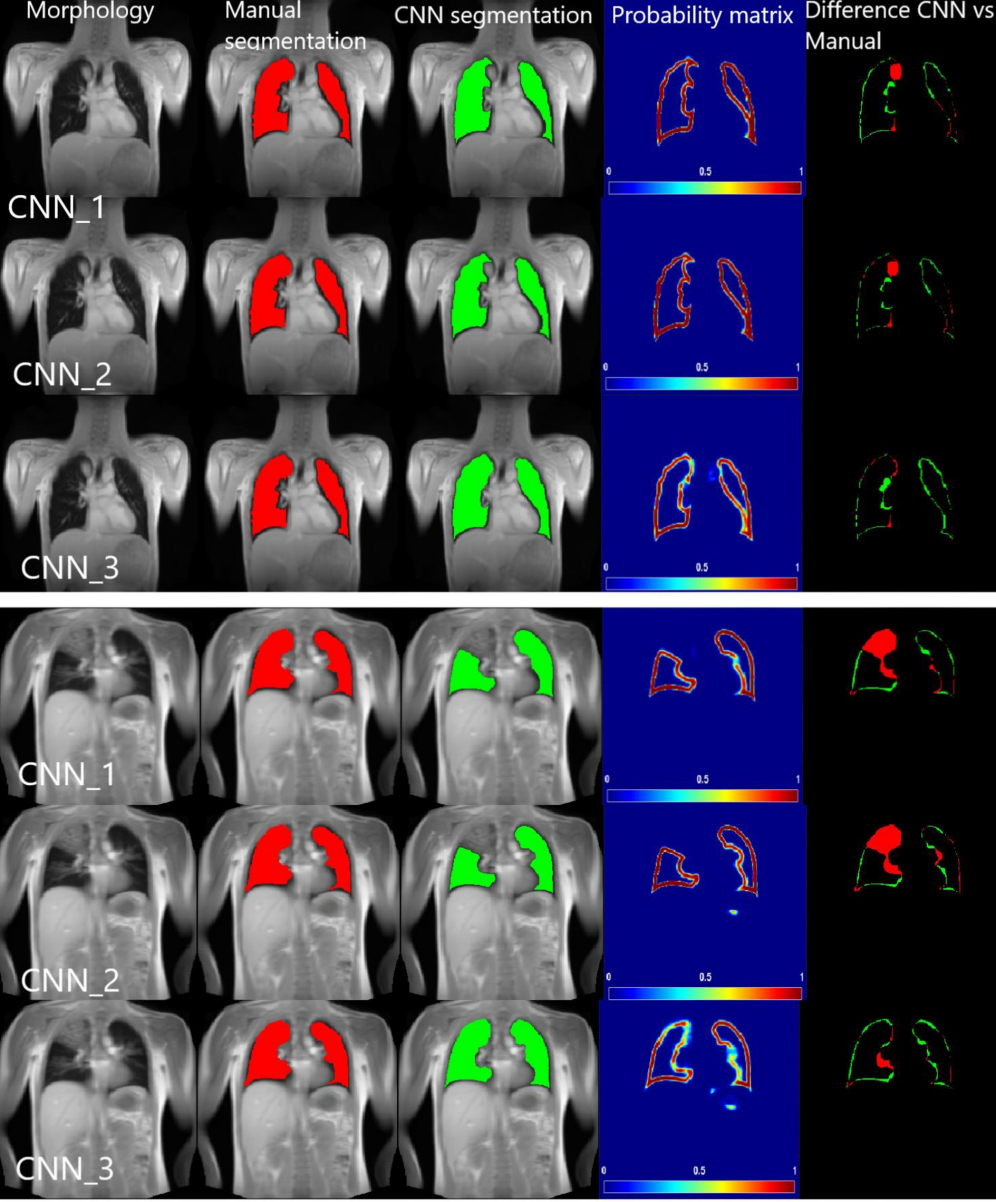
Exemplary segmentation results of CNN_Unbal/NoCons_, CNN_Bal/NoCons_ and CNN_Bal/Cons_ for two patients with CF. The first column represents the morphology image, the second column the manual segmentation, the third column the CNN segmentation after postprocessing. The fourth column represents the output of the CNN, the probability matrix. The fifth column represents the difference between manual and CNN segmentation.

A postprocessing algorithm was implemented to generate the final lung parenchyma segmentation from the contour’s probability matrix (see Fig 4 and fourth column). A threshold of 0.06% was empirically determined to obtain a closed lung contour. Finally, the lung contours were filled to obtain the final lung parenchyma segmentation, see Fig 3 third column. In addition, all algorithms except the training, were created in MATLAB 2020b (Matlab 2020b, MathWorks, Natick, MA, USA).

### Comparison of different training strategies

Three networks were trained to compare our different training strategies:

- CNN_Unbal/NoCons_: Training included the parameters mentioned above, without balanced augmentation and without artificially-generated consolidations. A conventional data augmentation, comprising rotations, translations and re-scaling, was performed in the 1666 image training set. Each image being augmented ten times (plus original data) results in a total training dataset of 18326 MR images with their corresponding ground truth.
- CNN_Bal/NoCons_: Training included the parameters mentioned above with a balanced augmentation but without artificially-generated consolidations.
- CNN_Bal/Cons_: Training included the parameters mentioned above with balanced augmentation and all input images had artificially-generated consolidations.

### Statistical analysis

The metrics used for the test datasets were the Sørensen-Dice (*SDC*) similarity coefficient (Eq 1), which is proportional to the intersection between two segmentations divided by the sum of these segmentations.


$$SDC(X,Y)=\frac{2|X\cap Y|}{\left|X|+|Y\right|}$$ (1)

where |*X*| and |*Y*| are number of elements in each sample. Additionally, the Hausdorff distance (*HD*) coefficient (Eq 2) was calculated.


HD(A,B)=max{min(A,B),min(B,A)} (2)

where *A* and *B* are two non-empty subsets of a metric space. A Student’s t-test (Eq 3) and Bonferroni correction on the *SDC* and *HD* was used to assess statistical differences between the three networks.


$$\text{t}=\frac{\hat{x}-\hat{y}}{\sqrt{\left(s_{1}^{2}/n_{1}\right)+\left(s_{2}^{2}/n_{2}\right)}}$$ (3)

where $\hat{x}$ and $\hat{y}$ are the sample means. $s_{1}^{2}$ and $s_{1}^{2}$ are the sample variances and *n*_1_ and *n*_2_ are the sample sizes. Finally, to avoid some Type I errors, the Bonferroni’s correction was performed so that the significance level of 0.05 was corrected to 0.016.

## Results

**Fig 5:**
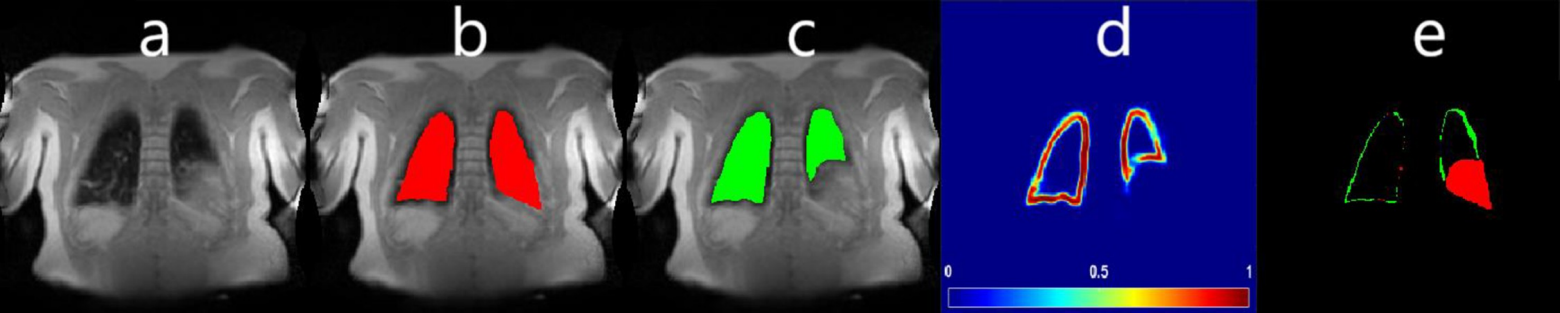
Exemplary failure segmentation result of CNN_Bal/Cons_ for a patient with CF. (a) morphological MR image. (b) the manual segmented lung parenchyma mask in red. (c) the segmented output result of CNN_Bal/Cons_ in green after the post-processing. (d) probability matrix of the output results of CNN_Bal/Cons_. (e) difference between the manual and automatic segmentation. In (e), the red colour represents the non-segmented pixels of the CNN and the extra segmented pixels of the CNN are shown in green.

Table 2 shows the SDC and HD values of the comparison of the three CNNs. After Bonferroni correction, for the 187 images of the test dataset without consolidations the *SDC* and *HD* of CNN_Bal/NoCons_ was significantly higher compared to CNN_Unbal/NoCons_ (*p*(*SDC*) = 0.0013 and *p*(*HD*) = 0.0012). The *SDC* and *HD* of CNN_Bal/Cons_ compared to the *SDC* of CNN_Unbal/NoCons_ was also significantly higher (*p*(*SDC*) = 0.0001 and *p*(*HD*) = 0.0009). The *SDC* and *HD* between CNN_Bal/NoCons_ and CNN_Bal/Cons_ was not significantly different (*p*(*SDC*) = 0.54 and *p*(*HD*) = 0.58). For the 38 images of the test dataset with consolidations, after Bonferroni correction, the *SDC* and *HD* of CNN_Bal/NoCons_ was not significantly different compared to CNN_Unbal/NoCons_ (*p*(*SDC*) = 0.53 and *p*(*HD*) = 0.56). The *SDC* and *HD* of CNN_Bal/Cons_ compared to CNN_Unbal/NoCons_ was significantly higher (*p*(*SDC*) = 0.0013 and *p*(*HD*) = 0.0148) and the SDC and *HD* between CNN_Bal/NoCons_ and CNN_Bal/Cons_ was also significantly higher (*p*(*SDC*) = 0.0146 and *p*(*HD*) = 0.0152). Exemplary segmentation results are depicted in Fig 4. One out of the 38 MR images with consolidations resulted in an inadequate segmentation of the proposed model, see Fig 5.

**Table 2:** Presentation of the statistic indicators of our model for the 187 images of the test dataset without consolidations and the 38 images of the test dataset with consolidations; the mean Sørensen-Dice similarity (*SDC* ± standard deviation), p-values distribution of the *SD* and the mean Hausdorff (*HD*) distance coefficient with the corresponding p-values distribution of the *HD*.

CNNs	Statistical parameter (mean) of the 187 test-images without consolidations			
	***SDC* (%)**	**p-Value**	***HD* (*mm*)**	**p-Value**
**CNN_Unbal/NoCons_ vs CNN_Bal/NoCons_**	92.0 ± 6.6 vs 94.0 ± 5.3	0.0013	7.1*mm* ± 4.5*mm*	0.0012
**CNN_Unbal/NoCons_ vs CNN_Bal/Cons_**	92.0 ± 6.6 vs 94.3 ± 4.1	0.0001	6.4*mm* ± 4.2*mm*	0.0009
**CNN_Bal/NoCons_ vs CNN_Bal/Cons_**	94.0 ± 5.3 vs 94.3 ± 4.1	0.54	10.3*mm* ± 6.2*mm*	0.58
	Statistical parameter (mean) of the 38 test-images with consolidations			
	***SDC* (%)**	**p-Value**	***HD* (*mm*)**	**p-Value**
**CNN_Unbal/NoCons_ vs CNN_Bal/NoCons_**	89.0 ± 7.1 vs 90.2 ± 9.4	0.53	9.8*mm* ± 7.9*mm*	0.56
**CNN_Unbal/NoCons_ vs CNN_Bal/Cons_**	89.0 ± 7.1 vs 94.3 ± 3.7	0.0013	7.2*mm* ± 4.7*mm*	0.0148
**CNN_Bal/NoCons_ vs CNN_Bal/Cons_**	90.2 ± 9.4 vs 94.3 ± 3.7	0.0146	6.7*mm* ± 5.8*mm*	0.0152

The probability matrices of the proposed models (Fig 4 and fourth column) show the final results between CNN_Bal/NoCons_ and CNN_Bal/Cons_ without post-processing. As shown, in the part of the lung containing consolidations, CNN_Bal/Cons_ can segment the contour of the lung accurately. Fig 4 (top) shows that the CNN_Bal/Cons_, unlike CNN_Bal/NoCons_, is sensitive to include a small consolidation in the upper border of the right lung. In accordance with the SDC and HD results, the example shows that the proposed model (CNN_Bal/Cons_), independently of post-processing, provides a more accurate segmentation.

**Fig 6:**
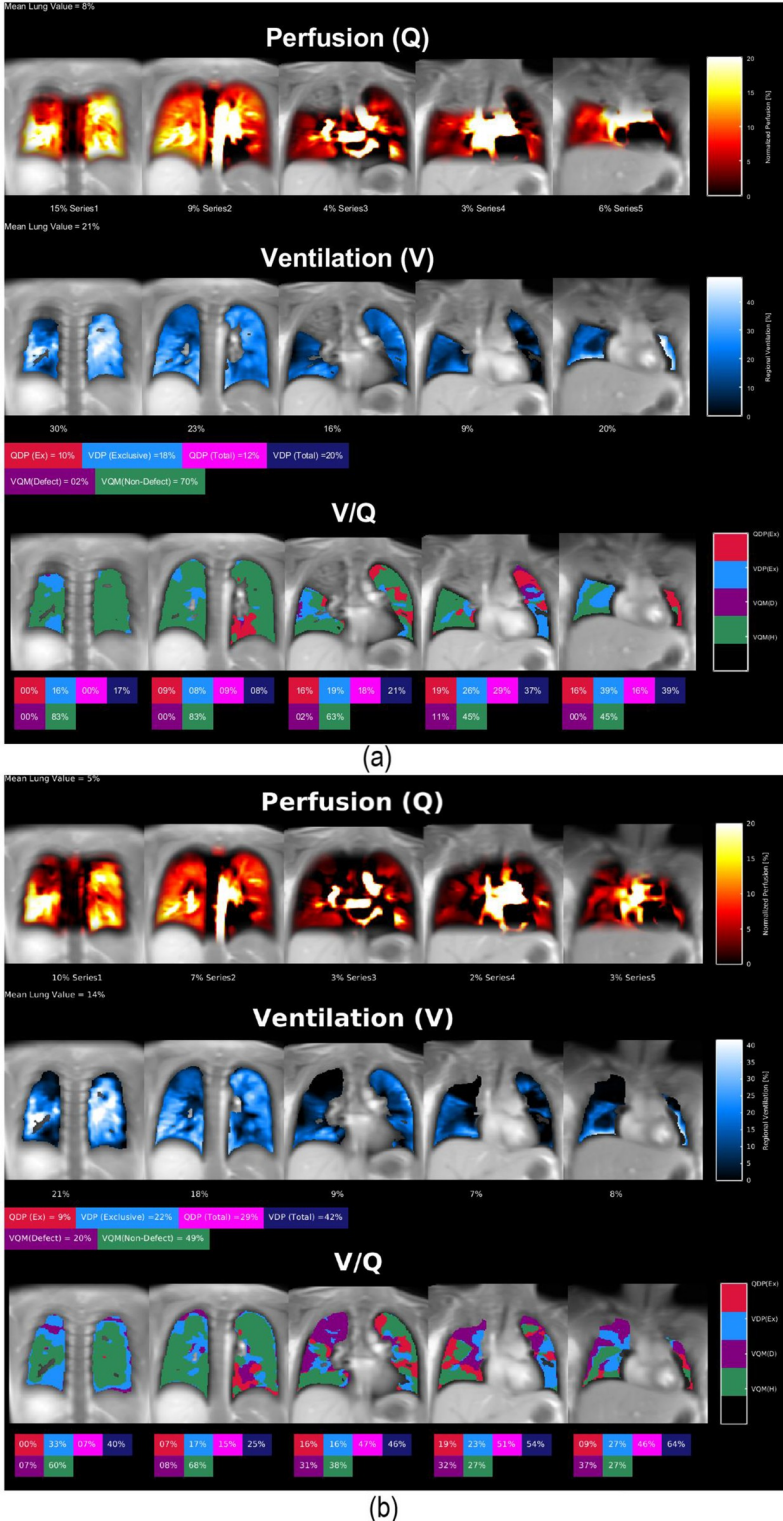
Impact on functional lung values caused by the improvements in segmentation performance: (a) shows the Perfusion (Q) and Ventilation (V) maps based on the segmentation from CNN_Bal/NoCons_: trained with balanced augmentation and without artificially-generated consolidations. (b) shows Q and V maps based on the segmentation from CNN_Bal/Cons_ (proposed method): trained with balanced augmentation with artificially-generated consolidations. Notice, that ventilation and perfusion parameters change essentially when whole lung parenchyma including the real consolidation is correctly segmented using CNN_Bal/Cons_. Ventilation and perfusion defect percentage maps increased substantially (from 20% up to 42% for ventilation and from 12% to 29% for perfusion) with the proposed CNN network. The images were created with Phase Resolved Functional Lung MRI^3^ (PREFUL) method.

Fig 6 shows the impact on functional lung values caused by the improvements in segmentation performance: the perfusion (Q) and ventilation (V) defect percentage maps based on the segmentation from CNN_Bal/NoCons_: trained with balanced augmentation and without artificially-generated consolidations and Q and V maps based on the segmentation from CNN_Bal/Cons_ (proposed method): trained with balanced augmentation with artificially-generated consolidations.

## Discussion

The main findings of this study are as follows:

1. Balanced augmentation in the training phase (CNN_Bal/NoCons_) improved segmentation results in comparison with CNN_Unbal/NoCons_ trained without balanced augmentation, and
2. artificially-generated consolidations in the training phase improved lung parenchyma segmentations results in patients with real consolidations.

The presented results confirm the hypothesis, that both proposed training procedures, including artificially-generated consolidations and a balanced data augmentation strategy, provide an increased segmentation quality in cases with visible lung parenchyma pathologies.

Most applications in the detection of pulmonary diseases applying Deep Learning are focused on CT data [27]. However, several studies on lung MR images applying CNNs have been presented. Tustison et al. proposed a lung segmentation CNN with template-based data augmentation strategy to quantify pulmonary ventilation defects from hyperpolarized helium [28] reaching a *SDC* of 94 ± 2% for the parenchyma segmentation. Willers et al. proposed an automatic artificial neural network (ANN) lung segmentation model for quantitative outcomes from functional pulmonary MRI, nevertheless, cases with consolidations or atelectasis were excluded from the final analyses [11]. Guo et al. proposed an semi-automated lung registration and segmentation [29] algorithm for proton-based ventilation MRI in free-breathing pulmonary ^1^H MR image processing to generate ventilation-defect-percent (VDP). A *SDC* of 95 ± 1.5% for lung segmentation was acquired. Nevertheless, is the application was limited to a balanced steady-state free precession sequence and tested only in patients with asthma.

Deep learning based synthetic image generation is a powerful emerging technology. Such image generation needs a training dataset with structures (in our case consolidations) that are supposed to be generated. There are generally two approaches on synthesizing training data for neural networks. A model-based approach where an algorithm is designed and engineered to synthesize data as specified. This requires no training data but cannot be generalized [30]. In a learning based approach, a model is trained on existing data and enabled to synthesize new data in the same space that it is trained on. These models generate excellent data as well but require data [31]. Since not enough data with consolidations are available at our site, the initial data were generated using the proposed method. The generation and implementation of synthetic data as a form of data augmentation has been also presented by Shin et al. [32], where final results demonstrated that synthetic data in the training phase improves the final segmentation results. Although the work of Shin et al. corresponds to brain tumor segmentation, the synthetic generation data method is comparable in the performance as observed for the method presented here.

### Limitations

Our study did have some important limitations. The segmentation of vessels was not implemented due to the low spatial resolution of the MR images. Thus, the segmented lung parenchyma will include smaller vessels. These vessels could influence the final evaluation of the lung parameters as explained by Winther et al. [33]. Therefore, for applications, which require a more accurate segmentation, further post-processing steps might be necessary.

Although, the training and test of the CNN included quite heterogeneous data, comprising two main sequences (FLASH and bSSFP) and also two widespread field strengths (1.5T and 3T). However, data from other sequences, data with broader spectrum of lung disease, data from low field MRI or a neonate application might be a challenge for the presented CNN.

Recently, a reformation o of 2D images was used to for 3D lung lobe segmentation [34]. Since 2D CNNs do not consider adjacent consecutive slices for the segmentation, more accurate lung parenchyma or lung lobe segmentation of 3D data is probably achieved with 3D CNNs. 3D imaging can extract more features and surrounding information that might be helpful for more precise lung parenchyma segmentation. Previously, other authors on segmenting of 3D morphological images in functional 3D pulmonary imaging [35]. The choice of using of 2D or 3D approach depends on the application. Since our workflow works with single slice data only, a 3D segmentation was not preferable in this case.

Also, when pixels in the structure of the lung closely match the pixels in the diaphragm, the CNN was not able to correctly segment this image, see Fig 5. However, even for manual segmentation it was difficult in this specific case to decide on the correct lung boundary. Therefore, regardless of the high rate of accurate segmentations, a human expert control is still essential, especially in the clinical context. Nevertheless, the supervision of automatically completed segmentations is still likely requiring much less time compared to manual segmentation.

## Conclusion

Accuracy of lung segmentation of 2D datasets can be improved by balanced augmentation of different anatomical slice positions and by including slices with artificially-generated consolidations. This is an important step to create robust automated lung MRI analysis pipelines, which will be integrated into the clinical workflow in the near future.

## Supporting information

S1 File (DOCX)
