# Supplementary material for: Artificially-generated consolidations and balanced augmentation increase performance of U-net for lung parenchyma segmentation on MR images
Source: PLoS One. 2023 May 9;18(5):e0285378. doi: 10.1371/journal.pone.0285378 (PMC10168553; doi:10.1371/journal.pone.0285378)
Supplement: S1 File — (DOCX) [file pone.0285378.s001.docx]

| Funding | The authors received no specific funding for this work. |
| --- | --- |
| Competing interests | The authors have declared that no competing interests exist. |
| Acknowledgments | The authors would like to express their gratitude to the medical technical assistants from the Department of Radiology for their support with the MR measurements. |
